# A unique set of 6 circulating microRNAs for early detection of non-small cell lung cancer

**DOI:** 10.18632/oncotarget.9363

**Published:** 2016-05-14

**Authors:** Ann Rita Halvorsen, Maria Bjaanæs, Marissa LeBlanc, Are M. Holm, Nils Bolstad, Luis Rubio, Juan Carlos Peñalver, José Cervera, Julia Cruz Mojarrieta, Jose Antonio López-Guerrero, Odd Terje Brustugun, Åslaug Helland

**Affiliations:** ^1^ Department of Cancer Genetics, Institute for Cancer Research, OUS Radiumhospitalet, Oslo, Norway; ^2^ Department of Oncology, OUS Radiumhospitalet, Oslo, Norway; ^3^ Oslo Centre for Biostatistics and Epidemiology, Research Support Services, Oslo University Hospital, Oslo, Norway; ^4^ Department of Respiratory Medicine, OUS Rikshospitalet, Oslo, Norway; ^5^ Department of Medical Biochemistry, OUS Radiumhospitalet, Oslo, Norway; ^6^ Laboratory of Molecular Biology, Fundación Instituto Valenciano de Oncología, Valencia, Spain; ^7^ Department of Thoracic Surgery, Fundación Instituto Valenciano de Oncología, Valencia, Spain; ^8^ Department of Radiology, Fundación Instituto Valenciano de Oncología, Valencia, Spain; ^9^ Department of Pathology, Fundación Instituto Valenciano de Oncología, Valencia, Spain

**Keywords:** circulating microRNAs, biomarker, lung cancer, early detection, COPD

## Abstract

**Introduction:**

Circulating microRNAs are promising biomarkers for diagnosis, predication and prognostication of diseases. Lung cancer is the cancer disease accountable for most cancer deaths, largely due to being diagnosed at late stages. Therefore, diagnosing lung cancer patients at an early stage is crucial for improving the outcome. The purpose of this study was to identify circulating microRNAs for detection of early stage lung cancer, capable of discriminating lung cancer patients from those with chronic obstructive pulmonary disease (COPD) and healthy volunteers.

**Results:**

We identified 7 microRNAs separating lung cancer patients from controls. By using RT-qPCR, we validated 6 microRNAs (miR-429, miR-205, miR-200b, miR-203, miR-125b and miR-34b) with a significantly higher abundance in serum from NSCLC patients. Furthermore, the 6 miRNAs were validated in a different dataset, revealing an area under the receiver operating characteristic curve of 0.89 for stage I-IV and 0.88 for stage I/II.

**Materials and Methods:**

We profiled the expression of 754 unique microRNAs by TaqMan Low Density Arrays, and analyzed serum from 38 patients with NSCLC, 16 patients suffering from COPD and 16 healthy volunteers from Norway, to explore their potential as diagnostic biomarkers. For validation, we analyzed serum collected from high-risk individuals enrolled in the Valencia branch of the International Early Lung Cancer Action Program screening trial (n=107) in addition to 51 lung cancer patients.

**Conclusion:**

Considering the accessibility and stability of circulating miRNAs, these 6 microRNAs are promising biomarkers as a supplement in future screening studies.

## INTRODUCTION

Worldwide, lung cancer is the primary cause of cancer death. Non-small cell lung cancer (NSCLC) represents approximately 85% of all diagnosed lung cancer cases, and adenocarcinomas and squamous cell carcinomas are the main histological subtypes. Despite improvements in targeted therapies, the overall survival rates remain poor, which is partly explained by delayed diagnosis. Only 25% of the patients are diagnosed at an early stage, when the tumor is accessible for curative surgery [1]. Today, several low-dose computed tomography (LDCT) screening studies have been initiated to evaluate the effect of such programs on lung cancer mortality. One concern of using annual CT scanning is that it may lead to harm due to radiation exposure [2]. Another concern of using LDCT as a screening tool is the high risk for false positives. A recently published meta-analysis of LDCT screening reported 235 false positive nodules detected for every 1000 individuals screened [3]. In the National Lung Screening Trial (NLST), 24.2% of enrolled persons at risk screened with LDCT had a positive result. Out of these, 96.4% were false positive. This, in turn, can lead to unnecessary and potentially harmful further diagnostics. However, a reduction in mortality of 20% was observed when screened with LDCT, which indicates that screening is beneficial [4]. Since a blood test is readily accessible, circulating biomarkers for early detection of lung cancer may be an integrated part of future lung cancer screening programs.

MicroRNAs are small non-coding oligonucleotides with capacity to negatively regulate expression of mRNAs by inhibition of protein translation [5]. MicroRNAs are involved in several, essential biological processes, and based on microRNA profiles, tumor cells can be distinguished from normal cells [6]. Interestingly, tumor cells can release microRNAs into circulation which can be detected in fluids like blood [7]. These microRNAs are shown to be remarkably stable and are able to avoid degradation from RNases. In blood, microRNAs can be incorporated into exosomes or bound to proteins like Argonaut [8] or HDL and transported via serum to a target cell with possible gene expression alteration [9]. Much effort has been invested in order to find circulating microRNAs for cancer identification, treatment monitoring and prognostication. Several studies have reported evidence of an altered circulating microRNA expression profile in cancer patients, including patients with breast cancer and lung cancer [10, 11]. However, due to small cohorts, lack of validation and use of different types of microRNA screens, it has proved challenging to reproduce initially promising discoveries within this field.

The purpose of this study was to find circulating biomarkers specific for lung cancer, capable of discriminating lung cancer patients from those with chronic obstructive pulmonary disease (COPD) and healthy normal volunteers.

## RESULTS

By using TLDA cards, a total number of 754 microRNAs were screened in serum from lung cancer patients, COPD patients, and healthy volunteers. Due to low abundance of microRNAs in serum, we did a manual inspection of the expression curve of each microRNA. Those with irregular shape of the curve, or microRNAs expressed in less than 30% of the samples were removed. 272 microRNAs passed the abovementioned control-steps, and were used in further analyses. Many of the microRNAs found in blood showed similar expression profile in both groups. In order to capture microRNAs differentially expressed, we performed a PAM analysis, resulting in a list of 37 significantly differentially expressed microRNAs (FDR<0.01) (Supplementary Table 1).

For visualization, a supervised hierarchical clustering based on the 37 microRNAs identified two main microRNA clusters, as displayed in Supplementary Figure 1. In order to find a signature able to distinguish lung cancer patients from controls, microRNAs with a higher abundance in serum from lung cancer patients compared with the controls were further examined. Out of the 37 microRNAs identified by PAM analysis, 10 were upregulated (miR-205, miR-200b, miR-125b, miR-34b, miR-429, miR-203, miR-186, miR-1180, miR-601 and miR-378). Due to low expression of circulating microRNAs, a technical validation was performed on the 10 selected microRNAs. Seven of these (miR-205, miR-200b, miR-125b, miR-34b, miR-429, miR-203, and miR-1180) showed a difference in expression between the two groups and were further validated in the external validation cohort. We did not find any correlation between tumor stage or tumor size and the level of microRNA's in the discovery set or in the validation set.

### Validation of selected microRNAs

The seven selected microRNAs were analyzed in the external validation set, using RT-qPCR and single assays. All microRNAs except miR-1180 showed a significantly higher abundance in serum from lung cancer patients compared with those with no detected tumor, and miR-1180 was left out of further analysis. The differences in expression are shown in Table 1.

**Table 1:** An independent samples t-test was conducted to compare expression of the 6 microRNAs for lung cancer patients and controls

Sample Set	miRNA	p-value	(t-test)	Mean difference	95% CI mean difference
**Discovery Set**	miR125bCt	0.025	−1.80	−3.36	−0.24
	miR200bCt	<0.001	−4.65	−6.35	−2.96
	miR203Ct	<0.001	−1.60	−2.35	−0.86
	miR205Ct	<0.001	−4.45	−6.12	−2.79
	miR34bCt	0.082	−1.48	−3.16	0.19
	miR429Ct	<0.001	−3.25	−4.89	−1.61
**Validation Set**	miR125bCt	<0.001	−2.38	−3.20	−1.57
	miR200bCt	<0.001	−1.46	−1.83	−1.09
	miR203Ct	<0.001	−2.08	−3.14	−1.02
	miR205Ct	<0.001	−2.56	−3.26	−1.87
	miR34bCt	0.002	−0.74	−1.20	−0.29
	miR429Ct	<0.001	−1.65	−2.13	−1.17

Data are shown for both the discovery set and validation set.

**Figure 1:**
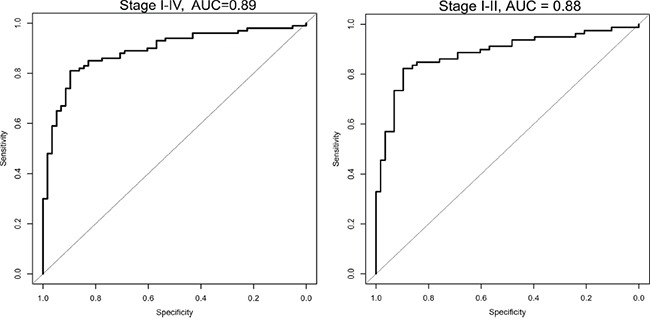
ROC Curve combined for the selected 6 microRNA analyzed in the validation set, for all stages (I-IV) and for only the early stages (I/II) The plot shows the sensitivity versus specificity. The area under the curve (AUC) gives a summary of the sensitivity and specificity across a range of different cut off points.

**Figure 2:**
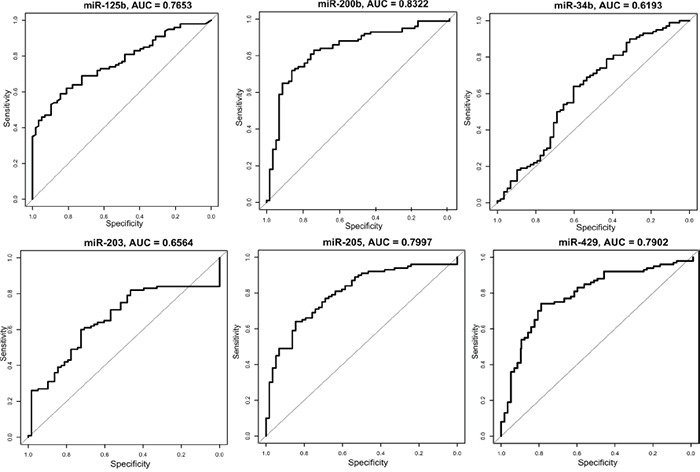
ROC curves for the 6 validated microRNAs analyzed in the validation set The plots show the sensitivity versus specificity. The area under the curves (AUC) is given for each microRNA.

In order to visualize the sensitivity and specificity for the six microRNAs combined, a ROC curve was calculated (Figure 1). To see how each microRNA performed individually, a ROC curve and the area under the curve (AUC) was calculated for the 6 validated microRNAs (Figure 2). With an AUC on 0.83, miR-200b revealed the highest sensitivity and specificity of the 6 microRNAs.

For the validation set, the cross-validation based area under ROC curve was 0.89 for the 6-microRNAs combined (logistic regression, the coefficients are shown in Supplementary Table 2). The sensitivity and specificity for the 6-miRNAs were obtained by a threshold value. With a threshold value of 0.37, a sensitivity of 88 % and a specificity of 71 % were found. Depending on clinical priorities, a higher sensitivity can be achieved by lowering the threshold. The pair-wise correlations for the six microRNAs ranged from 0.01 – 0.76, supporting their usefulness in the prediction model (Supplementary Table 3).

In order to emphasize the predictive potential of the six microRNAs in only the early stage patients, stage IIIa and IV were removed, and stage Ia/Ib/IIa/IIb were analyzed separately (logistic regression, Supplementary Table 2). For the 6 microRNAs combined an area under the ROC curve of 0.88 was achieved (Figure 1), and a sensitivity of 85% and a specificity of 74% were found.

### Does the microRNA profile in serum reflect the microRNA profile in tumor?

A study based on microRNA expression in lung adenocarcinoma samples and normal lung samples has been performed on the same patients by Bjaanæs and colleagues [12]. They identified 129 significantly differentially expressed microRNAs in lung adenocarcinoma tissue compared with adjacent normal lung tissue. When we compared the microRNA profile in tumor tissue with the serum profile, only 8 overlapping microRNAs were found. However, 3 of these were in the opposite direction (Supplementary Table 4).

## DISCUSSION

By comparing expression levels of serum microRNAs in lung cancer patients with serum from COPD patients and healthy controls, we identified 37 differentially expressed microRNAs. It is important to bear in mind that most of the measured circulating microRNAs in blood are released by other cells than tumor cells [13], Therefore, we have focused on the circulating microRNAs upregulated in lung cancer patients. With high sensitivity and specificity, six microRNAs distinguished individuals with lung cancer from those without. Focusing on only the early stages, the sensitivity and specificity remained high indicating that these microRNAs are able to capture lung cancer patients very early in the cancer development.

The 6 identified microRNAs are known to be involved in tumor progression. MiR-200b and miR-429 are both members of the miR-200 family, reported to be elevated in serum from many cancer types, including breast, ovarian and pancreatic cancer [14–16]. Independent studies have revealed that the miR-200 family and miR-205 play critical roles in regulating EMT by targeting the E-cadherin repressors ZEB1 and ZEB2 [17]. Furthermore, ZEB1 has been reported to repress miR-203 in the ZEB/miR-200 feedback loop [18]. In a recent study, the metastatic capacities of the miR-200s were investigated in breast cancer. The miR-200s were secreted in extracellular vesicles (EV) in human breast cancer cell lines, engulfed in non-metastatic cells, altering the gene expression and facilitating mesenchymal-to-epithelial transition (MET) [19]. Both miR-205 and miR-203 have been reported to be elevated in blood from NSCLC patients [20]. In some studies, miR-205 is described as a tumor suppressor, but others link this microRNA to processes of tumor initiation and progression of cancer [21]. However, several studies have demonstrated that the release of miR-205 is selectively packed into exosomes and secreted by lung cancer cells, and the level of circulating miR-205 decreases after lung tumor removal [22].

In this study, miR-125b was elevated in the serum samples of lung cancer patients. Published reports on miR-125b are somewhat contradictory, and it seems like this microRNA can act both as a tumor suppressor and have an oncogenic role in tumorigenesis [23, 24]. Nevertheless, miR-125b can directly target the tumor suppressor gene *TP53* [25] and the downstream modulator *TP53INP1* [26], contributing to malignancy. Interestingly, miR-34b, which is a transcriptional target of TP53 often found downregulated in tumor [27], showed a higher abundance in sera from lung cancer patients than in the control sera in this study. In another study of circulating microRNA, miR-34b was also found to be upregulated in sera of prostate cancer patients with respect to normal individuals [28].

### MicroRNAs for detection of lung cancer

Blood-based screening tests may be a crucial diagnostic tool to detect lung cancer at an early stage. Today, LDCT is widely used for tumor detection, and although it has been shown to reduce lung cancer deaths, the specificity is not optimal. A blood-based test with high sensitivity would be of great value as an addition to LDCT. This could increase both specificity and sensitivity. Since most screening projects have focused on the high-risk population of heavy smokers, we wanted to develop a blood-based screening test to be offered to all at risk. In order to capture lung cancer also among never smokers with a blood based test, 47% never-smokers were included in lung cancer group in the discovery cohort. However, since LDCT screening cohorts don't include never-smokers, and only 2 never-smokers were included in the Valencia-cohort, we were not able to confirm this potential in the external validation, and future studies need to address this further. On the other hand, six microRNAs were validated in a cohort slightly different from the discovery set, indicating the robustness of the identified microRNAs. Many researchers report on biomarkers with high potential for lung cancer detection [29, 30], but a commercial blood-based screening test is yet not available. Recently, a 4-miR serum based signature able to discriminate lung cancer patients from non-cancer controls with high AUC was identified. Two of the reported microRNAs (miR-141 and miR-200b) belong to the miR-200 family [31]. Interestingly, circulating miR-141, miR-203 and miR-200b have recently also been reported to discriminate between colorectal patients and healthy controls [32]. This is in line with our study, where miR-200b, miR-203 and miR-429 could discriminate between lung cancer and controls. However, most of the identified signatures reported are not consistent with other reported microRNA signatures. This may be due to many factors such as use of limited number of samples or pooled samples in the discovery set [30, 33, 34], or studies based on candidate markers [35, 36]. A promising plasma based microRNA ratio signature was tested in a screening trial revealing a high sensitivity and specificity when combined with LDCT. In that study, 1000 blood samples were collected, identifying 60 of 69 lung cancers and reducing the false positives down to 3.7% [37]. In the present study, we chose another strategy, analyzing serum samples using global normalization and focusing on only microRNAs with higher abundance in serum from lung cancer patients relatively to the controls. A reduction in microRNA abundance is most likely an unspecific reaction, and cannot be explained by the tumor itself [38]. In addition, we have a thorough handling of missing values, a substantial underestimated bias when analyzing low abundant molecules [39]. A lot of microRNAs cannot be detected in the blood due to biological features and probably not due to technical issues, and should be handled with a cut-off value and not being excluded from the analysis [39]. We replaced the missing values with the lowest value in the dataset on linear scale, before log2 transformation of the data. Here, 70 serum samples were used in the discovery set, 158 samples in the validation set, and 754 unique microRNAs were screened in the discovery set. However, this was explored mainly in adenocarcinomas and should be further investigated in all types of lung cancer histology. Due to tumor heterogeneity and differences between patients, tumors can act very differently and the regulation of tumor cells may vary radically between individuals [29]. We considered that using serum from COPD patients as part of the controls as extremely important in order to avoid a smoking based signature. It's also crucial to validate the findings in an independent cohort, and by confirming our results in the IELCAP study proves the robustness of our study. Two prerequisites for clinically effective biomarkers are high specificity and sensitivity. In our study, the combination of the six microRNAs revealed a higher AUC than any of the single microRNAs. One marker may detect only a portion of the patients, but by combining several biomarkers, the possibility of capturing a high number of the patients with increased diagnostic accuracy might be feasible.

### Can the microRNA profile in serum reflect the microRNA profile in tumor?

When we compared the microRNA profile found in tumor with the profile in serum, only 5 microRNAs were significantly differentially expressed in the same direction. Other studies have demonstrated that microRNA profile in serum does not mirror the microRNA profile found in tumor [28, 30]. This may indicate that microRNAs are not randomly shed from tumor, but selected for their ability to regulate important mRNAs. Therefore, microRNA candidates from tumor profiling may be inappropriate as biomarkers in serum.

## MATERIALS AND METHODS

### Serum samples

**Figure 3:**
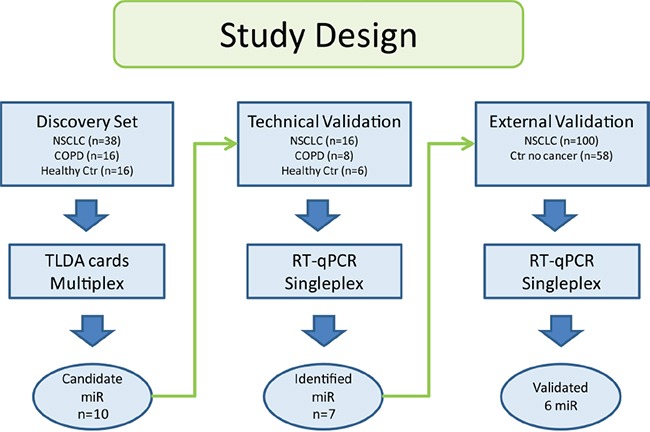
The study was divided into three phases Samples in the discovery set were used to find candidate microRNAs. Technical validation of the candidate microRNAs was performed on a subset of the same samples. Identified microRNAs from the discovery set were analyzed in the external validation.

In the discovery set, the patients were diagnosed with operable NSCLC from 2006 to 2011, and underwent curatively intended surgical resection at Rikshospitalet, Oslo University Hospital, Norway. The procedure for blood samples handling are outlined in Supplementary Table 5. The study was approved by the Regional Ethics Committee (S-05307), and written informed consent was obtained from all patients. Clinical data were collected from questionnaires, the medical journal, histology reports and follow up information from the patients’ local hospitals. All patients selected for the discovery cohort were diagnosed with adenocarcinoma. A predominance of stage I and II were included due to inoperable tumors in later stages. In order to capture the profile of the never smokers, 47.4% of the lung cancer patients were never smokers. For all selected patients, microRNA profiling in tumor has previously been performed by Bjaanæs and colleges [12]. Sera from 16 patients diagnosed with COPD and 16 healthy volunteers were used as controls [40]. The overview of the study design is displayed in Figure 3 and the clinical data are shown in Table 2.

**Table 2:** Information about gender, stage, age and smoking status for all the patients in this study are shown in the table

	Discovery Set				Validation Set		
	NSCLC	COPD	Healthy Control	P value	NSCLC	Healthy Control	P value
	n = 38 No.(percent)	n = 16 No.(percent)	n = 16 No.(percent)		n = 100 No.(percent)	n = 58 No.(percent)	
Gender							
*Male*	8 (21)	8 (50)	7 (43.8)	p = 0.022†	72 (72)	34 (59)	
*Female*	30 (79)	8 (50)	9 (56.2)		28 (28)	24 (41)	p = 0.085†
Age(mean)	66.8	66.5	52.9	p = 0.017††	62.6	57.6	p < 0.001††
Smoking status							
*Never smoker*	18 (47.4)	0 (0)	3 (18.8)		2 (2)	0 (0)	
*Former/current smoker*	20 (52.6)	16 (100)	13 (81.2)		87 (87)	58 (100)	
*Unknown*				p = 0.001†	11 (11)		p = 0.519†††
Packyears mean	6.6*	ND	ND		43.8	30.8	p < 0.001††
Histology							
Adenocarcinomas	38 (100)				98 (98)		
Squamous cell carcinomas	0 (0)			NA	2 (2)		NA
Tumor stage							
*Ia/Ib*	24 (63.2)				65 (65)		
*IIa/IIb*	5 (13.2)				14 (14)		
*IIIa*	8 (21)				18 (18)		
*IV*	1 (2.6)			NA	2 (2)		NA

* 47% never-smokers included

† Pearson's Chi-square test

†† Students T-test

††† Fisher's Exact Test

NA: not assessed

ND: no data

Samples from 107 persons enrolled into the International Early Lung Cancer Action Program (IELCAP, Valencia branch), were obtained from the Biobank of the Fundación Instituto Valenciano de Oncología (FIVO). 58 of these had no cancer detected with CT, denoted controls, and 49 were diagnosed with lung cancer. These series corresponded to a high-risk population for lung cancer >50 years old, heavy smokers for more than 15 years but without any history of cancer. All participants were examined with CT and whole blood was collected before diagnostic scanning. In addition, 51 serum samples from lung cancer patients from the Thoracic surgery department in Valencia were analyzed.

### MicroRNA screening with TLDA cards

From 500μl serum, totalRNA was extracted using miRCURY™ RNA isolation kit for biofluids (cat#300113, Exiqon) according to manufactures protocol. TotalRNA (totRNA) quantity and quality (yield, 260/280 ratio and 260/230 ratio) were determined with the NanoDrop ND-1000 spectrophotometer (Thermo Scientific).

First strand synthesis was performed using megaplex™ RT primer pool (pool A and B, cat#4444745, Life Technologies) and TaqMan® MicroRNA reverse transcription kit (cat#4366596 Life Technologies). For each sample, 30 ng totRNA was added to the reaction mix. In order to increase the sensitivity, preamplification of the cDNA was implemented using megaplex™ preamp primer pool (pool A and B, cat#4444748, Life Technologies) and TaqMan® preamp master mix (cat#4391128, Life Technologies). The samples were diluted with 75μl of 0.1XTE pH 8.0 and then added with TaqMan® Universal PCR Master Mix, NoAmpErase® UNG, 2X. The reaction mix was applied to the TaqMan® Low Density Arrays (TLDA; Life Technologies, cat# 4444913) and loaded onto the 7900HT Fast thermocycler system (Life Technologies) for analysis. The protocol from supplier (PN 4399721) was followed for all the procedures. The microRNA microarray data have been deposited in the Gene Expression Omnibus (GEO) (http://www.ncbi.nlm.nih.gov/geo) with the accession number GSE70080.

### Validation with reverse transcription-quantitative PCR (RT-qPCR)

Due to low expression levels of microRNAs in sera, a technical validation was performed with RT-qPCR for the ten candidate microRNAs upregulated in sera from lung cancer patients. Seven of these were further evaluated in the independent screening cohort of serum samples, using the same technique. The microRNAs were first reverse transcribed (MicroRNA reverse transcription kit, Life Technologies) and then quantified using qPCR. The procedures were performed according to standard protocols. The primers, reaction mix components and temperature conditions used for RT and qPCR are listed in Supplementary Table 6. All samples were run in triplicates. The comparative threshold (Ct) was used to evaluate the relative detection level of each microRNA.

### Normalization

The raw C_T_ data were exported to the ExpressionSuite software (v.1.0.3, Life Technologies) for normalization and quality control. Threshold and baseline was automatically calculated for each assay, and a global normalization was performed, as is recommended for large scale microRNA expression profiling. Global normalization first finds the common microRNAs among all samples. The median CT of those microRNAs is used as the normalizer, on a per sample basis [41]. Normalized ΔC_T_ data were used for calculation of relative gene expression in fold change (2 ^−ΔΔCt^) [42]. In the ExpressionSuite software a calculation score for the best choice of endogenous controls was performed. The three most uniformly expressed microRNAs (miR-220, miR-19b, sU6) from the TLDA cards were analyzed and chosen as reference genes for normalization in the validation set. To obtain the ΔC_T_, the mean expression level of the three microRNAs (C_T ref_) was subtracted from the mean Ct level (C_T target_) of each sample. Due to low expression of microRNAs in serum, missing values were most likely the results of too few RNA-copies. Not detected measurements were replaced by zero in linear scale. For the logarithmic scale the lowest expressed value for each microRNA replaced the missing values. Preamplification was not utilized in the validation and Ct values of <40 indicated the presence of microRNA.

### Statistical analysis

The dataset was divided into two groups consisting of 1) lung cancer patients 2) COPD/healthy controls, and a Prediction Analysis of Microarrays (PAM) was performed in R, ver. 2.12.2 [43]. The “nearest shrunken method” was used to identify those genes best characterizing each class [44]. Unsupervised hierarchical clustering was utilized in J-express [45]. A logistic regression model was built in the external validation set using 6 microRNAs as predictors and case/control status as the outcome. Leave-one-out-cross-validation was performed in R to assess how well the given model will generalize to an independent data set and a receiver operating characteristic curve (ROC) was generated from the cross-validation predictions. Chi-square test (*χ^2^*) and Student T-test were used to evaluate the frequency of clinical variables among lung cancer patients and controls (IBM SPSS Statistics, Version 21.0. Armonk, NY: IBM Corp).

## CONCLUSION

A global profiling of microRNAs in serum of lung cancer patients and controls was conducted and a panel of 6-microRNAs was identified in a Norwegian cohort. The microRNAs found in this study showed a significantly higher abundance in sera from lung cancer patients, compared with sera from COPD patients and healthy controls. Validation in a larger independent cohort from Spain confirmed a high sensitivity and specificity, which indicate that these six circulating microRNAs have a great potential to detect early stage lung cancer. Further validation of the six microRNAs identified in this study should be a part of a prospective study for early diagnosis of lung cancer.

## SUPPLEMENTARY FIGURE AND TABLES


