# Supplementary material for: A unique set of 6 circulating microRNAs for early detection of non-small cell lung cancer
Source: Oncotarget. 2016 May 14;7(24):37250–9. doi: 10.18632/oncotarget.9363 (PMC5095073; doi:10.18632/oncotarget.9363)
Supplement: Supplementary file 1 [file oncotarget-07-37250-s001.pdf]

A unique set of 6 circulating microRNAs for early detection of non-small cell lung cancer

SUPPLEMENTARY FIGURE AND TABLES

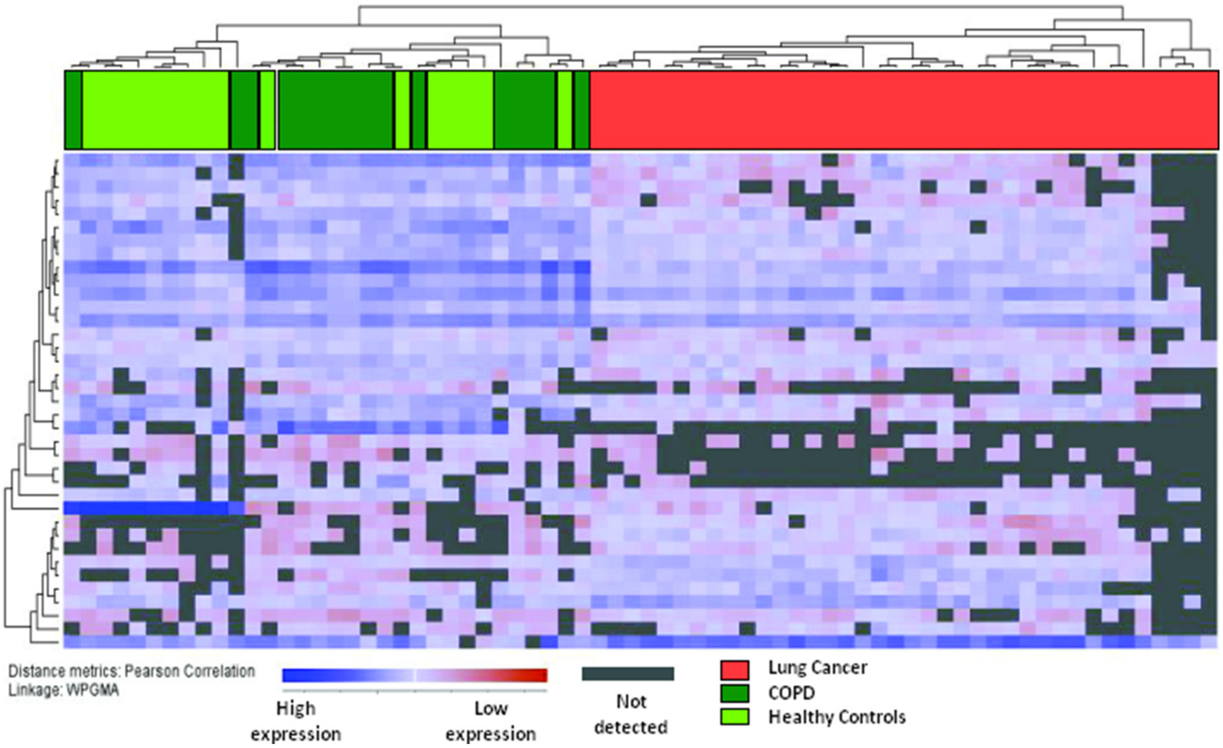

Supplementary Figure S1: Hierarchical clustering of the 37 microRNAs differentially expressed between lung cancer patients (red) and controls (green). On the left side of the heatmap, two clusters of microRNAs are seen. The microRNAs in the upper cluster showed a low abundance in lung cancer patients compared with COPD patients and healthy controls. The 10 microRNAs found in the lower cluster showed a higher abundance in lung cancer patients. In the heatmap, blue spots indicate high levels of microRNA, pink spots are low levels, and the grey spots are microRNAs not detected.

Supplementary Table S1: List of 37 significantly differentially expressed miRNAs (FDR=0.01)

	ID	p-value t-test	Fold Change	Express. serum (cancer)		ID	p-value t-test	Fold Change	Express. serum (cancer)
[1,]	hsa-miR-214	5.50E-22	125.386	Down	[20,]	hsa-miR-545	0.003392	23.545	Down
[2,]	hsa-miR-1285	5.18E-16	74.143	Down	[21,]	hsa-miR-548d-5p	0.0363388	6.896	Down
[3,]	mmu-miR-134	1.71E-11	12.301	Down	[22,]	hsa-miR-548J	0.0008765	365.573	Down
[4,]	hsa-miR-29c	2.06E-14	28.82	Down	[23,]	hsa-miR-423-5p	2.19E-07	5.315	Down
[5,]	hsa-let-7a	3.93E-08	9.043	Down	[24,]	hsa-miR-125b	1.48E-09	-3.806	Up
[6,]	hsa-miR-205	0.0014706	-8.747	Up	[25,]	hsa-let-7f	0.1172602	5.343	Down
[7,]	hsa-miR-184	4.79E-14	11.418	Down	[26,]	hsa-miR-18a	2.42E-07	3.395	Down
[8,]	hsa-miR-148b	2.34E-12	4.228	Down	[27,]	hsa-miR-186	9.89E-07	-11.678	Up
[9,]	hsa-miR-130a	4.11E-09	4.204	Down	[28,]	hsa-miR-1180	2.38E-13	-48.108	Up
[10,]	hsa-miR-200b	8.34E-05	-11.941	Up	[29,]	hsa-miR-381	0.5850876	4.568	Down
[11,]	hsa-miR-132	4.38E-16	22.983	Down	[30,]	hsa-miR-142-5p	2.60E-11	4.312	Down
[12,]	mmu-miR-495	1.90E-10	7.022	Down	[31,]	hsa-miR-203	7.06E-05	-1.894	Up
[13,]	hsa-miR-378	0.0028767	-1.878	Up	[32,]	mmu-miR-140	1.39E-06	2.814	Down
[14,]	hsa-miR-590-3P	6.80E-08	6.608	Down	[33,]	hsa-miR-34b	1.54E-06	-2.887	Up
[15,]	hsa-miR-210	2.24E-19	53.712	Down	[34,]	hsa-miR-376c	9.03E-06	4.41	Down
[16,]	hsa-let-7e	4.22E-09	7.599	Down	[35,]	hsa-miR-195	0.0128161	2.697	Down
[17,]	hsa-miR-429	0.0016299	-4.885	Up	[36,]	hsa-miR-26a	7.34E-05	2.473	Down
[18,]	hsa-miR-1300	0.0143191	8.336	Down	[37,]	hsa-miR-519b-3p	0.0180488	7.599	Down
[19,]	hsa-miR-601	5.65E-08	-3.155	Up					

The p-values (t-test) and fold changes are calculated for each miRNA. Expression level of miRNA in serum from cancer patients are specified as up or down, compared with controls

Supplementary Table S2: Coefficients and significance for the different miRNAs in the multivariate logistic regression model

A. Stage I-IV	Estimate	Standard Error	z value	Pr(>|z|)
**Mir125bCt**	-0.61196	0.20374	-3.004	0.00267
**Mir200bCt**	-0.70826	0.2757	-2.569	0.0102
**Mir203Ct**	0.15242	0.13294	1.146	0.25159
**Mir205Ct**	-0.1888	0.16654	-1.134	0.25693
**Mir34bCt**	-0.05477	0.19162	-0.286	0.77502
**Mir429Ct**	-0.93376	0.23035	-4.054	5.04E-05
**Intercept**	5.81644	1.08571	5.357	8.45E-08
**B. Stage I-II**	**Estimate**	**Standard Error**	**z value**	**Pr(>|z|)**
**Mir125bCt**	-0.56696	0.19891	-2.850	0.00437
**Mir200bCt**	-0.65911	0.27346	-2.410	0.01594
**Mir203Ct**	0.07711	0.13459	0.573	0.56668
**Mir205Ct**	-0.14427	0.17063	-0.846	0.39782
**Mir34bCt**	-0.02313	0.20756	-0.111	0.91126
**Mir429Ct**	-0.97141	0.24325	-3.993	6.51e-05
**Intercept**	5.64116	1.12070	5.034	4.81e-07

A is calculated for stage I-IV, and B is calculated for stage I-II.

Supplementary Table S3: Correlation matrix of the six microRNAs included in the prediction model

	miR125bCt	miR200bCt	miR203Ct	miR205Ct	miR34bCt	miR429Ct
miR125bCt	1	0.207	0.757	0.686	0.341	0.011
miR200bCt	0.207	1	0.154	0.533	0.344	0.617
miR203Ct	0.757	0.154	1	0.577	0.380	0.036
miR205Ct	0.686	0.533	0.577	1	0.420	0.329
miR34bCt	0.341	0.344	0.380	0.420	1	0.196
miR429Ct	0.011	0.617	0.036	0.329	0.196	1

Supplementary Table S4: When comparing the microRNA expression in tumor with the profile found in serum, 8 microRNAs were overlapping

miRNA	Expr. Serum	Expr. Tumor
hsa-miR-29c	down	down
hsa-let-7a	down	down
hsa-miR-130a	up	down
hsa-miR-210	down	up
hsa-miR-125b	up	down
hsa-let-7f	down	down
hsa-miR-195	down	down
hsa-miR-26a	down	down

The direction of expression for 3 of these microRNAs was in different direction in tumor compared with serum (shown in grey).

Supplementary Table S5: Procedures for blood sample handling

	Blood tubes	Collected	Centrifugation force / duration	Stored until RNA isolation
**Discovery set**	Vacuette with serum clot activatior	before surgery	2400rcf /12 min	minus 80°C
**Validation set**	Vacuette with serum clot activatior	before CT scanning	1811rcf/10 min	minus 80°C

Supplementary Table S6: A: RT Reaction mix components. B: Real Time PCR reaction mix components. C: Reverse transcription conditions. D: primer sequences for the different miRNAs

**A. Reverse Transcription**		
Component Master mix volume 10 µl reaction	1x µl	
100mM dNTPs (with dTTP)	0.10	
MultiScribe™Reverse Transcriptase, 50 U7µL	0.67	
10x Reverse Transcription Buffer	1.00	
Rnase Inhibitor, 20 U/µL	0.13	
Nuclease-free water	2.80	
Total volume	4.70	
3µl RT primer (spesific for each miRNA)(5X)	2.00	
3.3µl sample (30ng)	3.30	
**B. Real Time PCR**		
Master mix volume 10µl	1x	
TaqMan®Small RNA Assay (20x)	0.50	
Product from RT reaction	0.70	
TaqMan®Universal PCR Master Mix(2x),noUNG	5.00	
Nuclease-free water	3.80	
Total volume	10.00	
**C. Reverse Transcription Reaction (30ng totRNA input)**		
**Step type**	**Times (minutes)**	**Temp**
Hold	30	16°C
Hold	30	42°C
Hold	5	85°C
Hold	∞	4°C
**D. Primer sequences**		
**MiRNA**	**assay ID**	**sequence**
miR-205	509	UCCUUCAUUCCACCGGAGUCUG
miR-125b	449	UCCCUGAGACCCUAACUUGUGA
miR-200b	2251	UAAUACUGCCUGGUAAUGAUGA
miR-34b	2102	CAAUCACUAACUCCACUGCCAU
miR-203	507	GUGAAAUGUUUAGGACCACUAG
miR-429	1024	UAAUACUGUCUGGUAAAACCGU
miR-1180	2847	UUUCCGGCUCGCGUGGGUGUGU
